# Insights into Eyestalk Ablation Mechanism to Induce Ovarian Maturation in the Black Tiger Shrimp

**DOI:** 10.1371/journal.pone.0024427

**Published:** 2011-09-07

**Authors:** Umaporn Uawisetwathana, Rungnapa Leelatanawit, Amornpan Klanchui, Juthatip Prommoon, Sirawut Klinbunga, Nitsara Karoonuthaisiri

**Affiliations:** 1 National Center for Genetic Engineering and Biotechnology (BIOTEC), National Science and Technology Development Agency, Klong Luang, Pathumthani, Thailand; 2 Center of Excellence for Marine Biotechnology, Faculty of Science, Chulalongkorn University, Bangkok, Thailand; Florida International University, United States of America

## Abstract

Eyestalk ablation is commonly practiced in crustacean to induce ovarian maturation in captivity. The molecular mechanism of the ablation has not been well understood, preventing a search for alternative measures to induce ovarian maturation in aquaculture. This is the first study to employ cDNA microarray to examine effects of eyestalk ablation at the transcriptomic level and pathway mapping analysis to identify potentially affected biological pathways in the black tiger shrimp (*Penaeus monodon*). Microarray analysis comparing between gene expression levels of ovaries from eyestalk-intact and eyestalk-ablated brooders revealed 682 differentially expressed transcripts. Based on Hierarchical clustering of gene expression patterns, Gene Ontology annotation, and relevant functions of these differentially expressed genes, several gene groups were further examined by pathway mapping analysis. Reverse-transcriptase quantitative PCR analysis for some representative transcripts confirmed microarray data. Known reproductive genes involved in vitellogenesis were dramatically increased during the ablation. Besides these transcripts expected to be induced by the ablation, transcripts whose functions involved in electron transfer mechanism, immune responses and calcium signal transduction were significantly altered following the ablation. Pathway mapping analysis revealed that the activation of gonadotropin-releasing hormone signaling, calcium signaling, and progesterone-mediated oocyte maturation pathways were putatively crucial to ovarian maturation induced by the ablation. These findings shed light on several possible molecular mechanisms of the eyestalk ablation effect and allow more focused investigation for an ultimate goal of finding alternative methods to replace the undesirable practice of the eyestalk ablation in the future.

## Introduction

Eyestalk ablation has been employed to induce reproductive maturation in crustacean [1]. While the ablation can induce ovarian maturation, it also jeopardizes growth, shortens molting cycle, increases energetic demands [2], and resulting in an eventual loss in egg quality and high mortality [3]. Accordingly, predictable maturation and spawning in captive shrimp without the eyestalk ablation is a long–term goal for the shrimp industry [4].

Albeit undesirable effects, the eyestalk ablation is currently unavoidable to solve a serious problem of poor reproductive maturation in captivity of an economically important black tiger shrimp (*Penaeus monodon*). The reproductive biology of both male and female of this animal still remains poorly understood. In female, according to the gonadosomatic index (GSI; ratio of gonad weight to body weight) and physiological changes [5], the ovarian development can be separated into four stages: pre-vitellogenic, vitellogenic, early cortical rod, and spent (late cortical rod). Unlike ovarian development in natural habitat, the ovarian maturation of *P. kerathurus* [6] and *P. monodon* [7] does not naturally reach the later stages of ovarian maturation in captivity.

Crustacean eyestalk is a location for the X-organ sinus gland which is important for neuroendocrine system [8], [9]. In the spiny lobster *Panulirus argus*, the sinus gland seemed to contain a heat-stable factor inhibiting gonadal maturation [10]. In *P. monodon*, one of the important hormones secreted from this gland is gonad-inhibiting hormone (GIH) which is known to inhibit synthesis of a yolk protein precursor, vitellogenin [11]. Besides the GIH evidence, another hypothesis suggests that removing eyestalk also reduces light intensity and thereby inducing ovarian maturation. In the banana prawn *P. merguiensis*, dim light was reported to favor ovarian maturation and spawning [12]. To date, the exact mechanism of eyestalk ablation on the ovarian maturation is not conclusive.

To avoid the use of eyestalk ablation, understanding molecular effects of the ablation is essential for finding alternative measures to trigger ovarian maturation without the undesirable effects from the ablation. DNA microarray is a transcriptomic approach to study responses from an organism to stimuli. This high-throughput technique provides a global picture of gene expression patterns. Previously, we have successfully constructed and employed cDNA microarrays to study ovarian and testicular development in *P. monodon* [13], [14], [15]. In this study, we employed the cDNA microarrays to shed light on molecular mechanism of the eyestalk ablation effects by comparing gene expression levels of ovaries from non-ablated and ablated female broodstock over the course of seven days after the ablation. In addition, biological pathway information was used to identify putatively important pathways affected by the eyestalk ablation. The reverse-transcriptase quantitative PCR analysis was performed to validate the gene expression results and emphasize the potential involvement of these genes in the mechanism of ovarian maturation induced by the eyestalk ablation.

## Results

### Induction of ovarian maturation by eyestalk ablation

**Figure 1:**
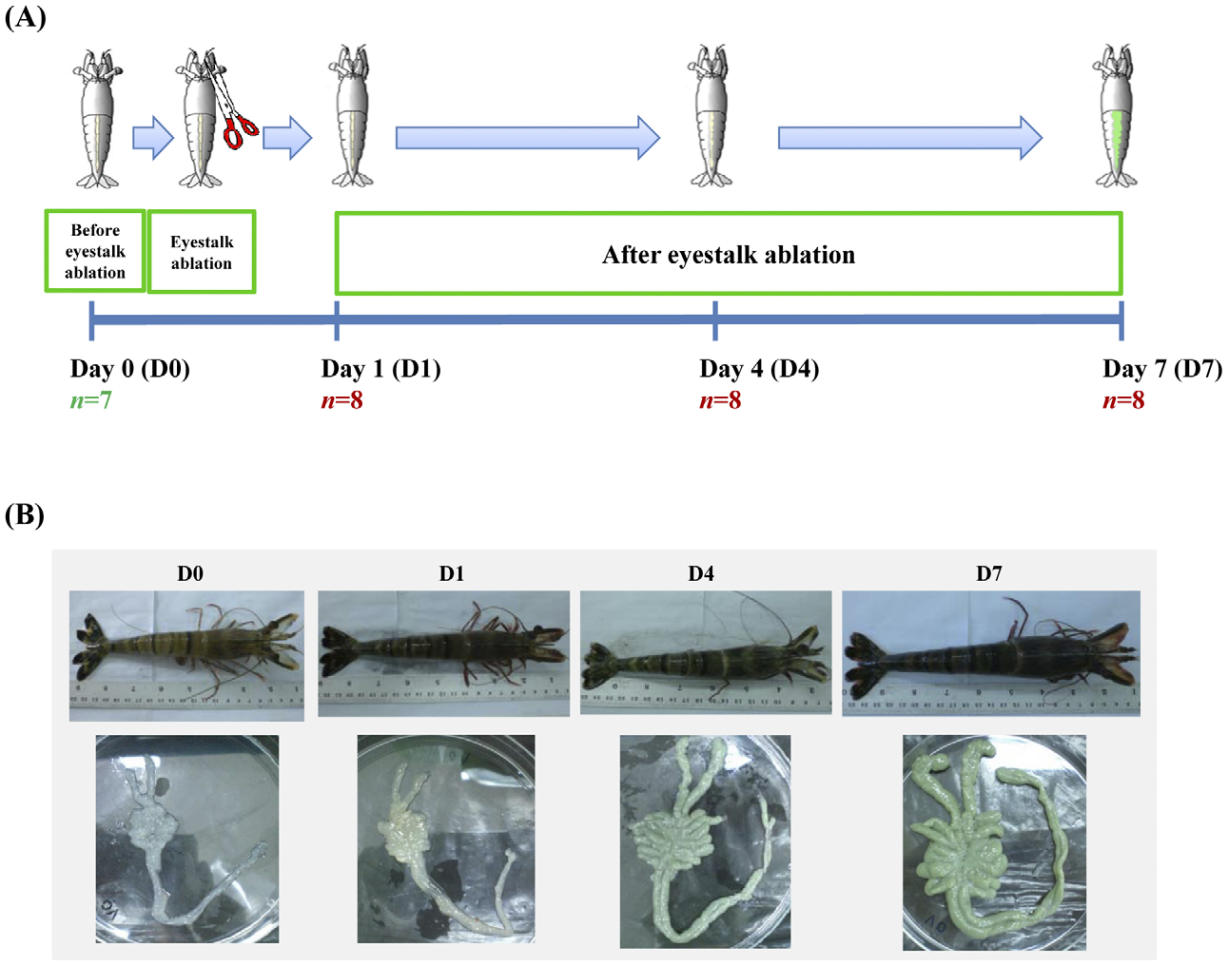
Diagram of the eyestalk ablation experiment and physiology of shrimp and ovary. (A) Eyestalk ablation experiment was conducted over 7 days. Ovary samples were collected before eyestalk ablation (D0) and after eyestalk ablation at Days 1, 4 and 7 (D1, D4, and D7, respectively). (B) Examples of representative shrimp (upper panel) and their ovaries (lower panel) from each time point.

To examine effects of eyestalk ablation on reproductive maturation of domesticated female black tiger shrimp broodstock at a molecular level, an eyestalk ablation experiment was conducted (Fig. 1A). Growth rates (length and body weight), ovary weight and gonadosomatic index (GSI) of the female shrimp significantly increased after the eyestalk ablation (Table 1). Length and body weight increased steadily over the eyestalk ablation time course and significantly increased from 17.3±0.5 cm and 62.6±5.5 g on Day 0 to 18.6±0.8 cm and 74.2±8.9 g on Day 7, respectively. Ovary weight increased from 0.7±0.2 to 3.6±1.9 g after seven days of the ablation. The significant increase in ovary weight during Day 4 to Day 7 indicated rapid maturation during that period after the ablation. In addition, GSI values, a known indicator of ovarian maturation in the female black tiger shrimp, significantly increased from 1.1±0.2% to 4.7±2.3% by Day 7. In addition to GSI values, the physiological changes and colors of ovaries indicated rapid maturation after the eyestalk ablation (Fig. 1B). The size and color of ovaries from the non-ablated shrimp were small and white, whereas those from the ablated shrimp increased in size and turned from white to yellow at Day 1, to light green at Day 4, and to dark green at Day 7 (Fig. 1B, lower panel).

**Table 1:** Summary of shrimp samples used in this study.

Shrimp Sample	Code	Number (*n*)	Length (cm)	Body Weight (g)	Ovary Weight (g)	GSI* (%)
Before eyestalk ablation	D0	7	17.3±0.5^a^	62.6±5.5^a^	0.7±0.2^a^	1.1±0.2^a^
After eyestalk ablation						
Day 1	D1	8	17.3±0.7^a^	63.1±7.7^a^	0.8±0.1^a^	1.2±0.1^a^
Day 4	D4	8	17.8±0.8^a,b^	64.3±8.1^a,b^	1.6±1.0^a^	2.5±1.8^a^
Day 7	D7	8	18.6±0.8^b^	74.2±8.9^b^	3.6±1.9^b^	4.7±2.3^b^

*Gonadosomatic Index (GSI) is calculated as a percentage of ovary weight by total body weight.

Different superscript letters indicate significant differences between groups (*p-*value<0.05, *Tukey* test).

### Global gene expression changes due to eyestalk ablation

**Figure 2:**
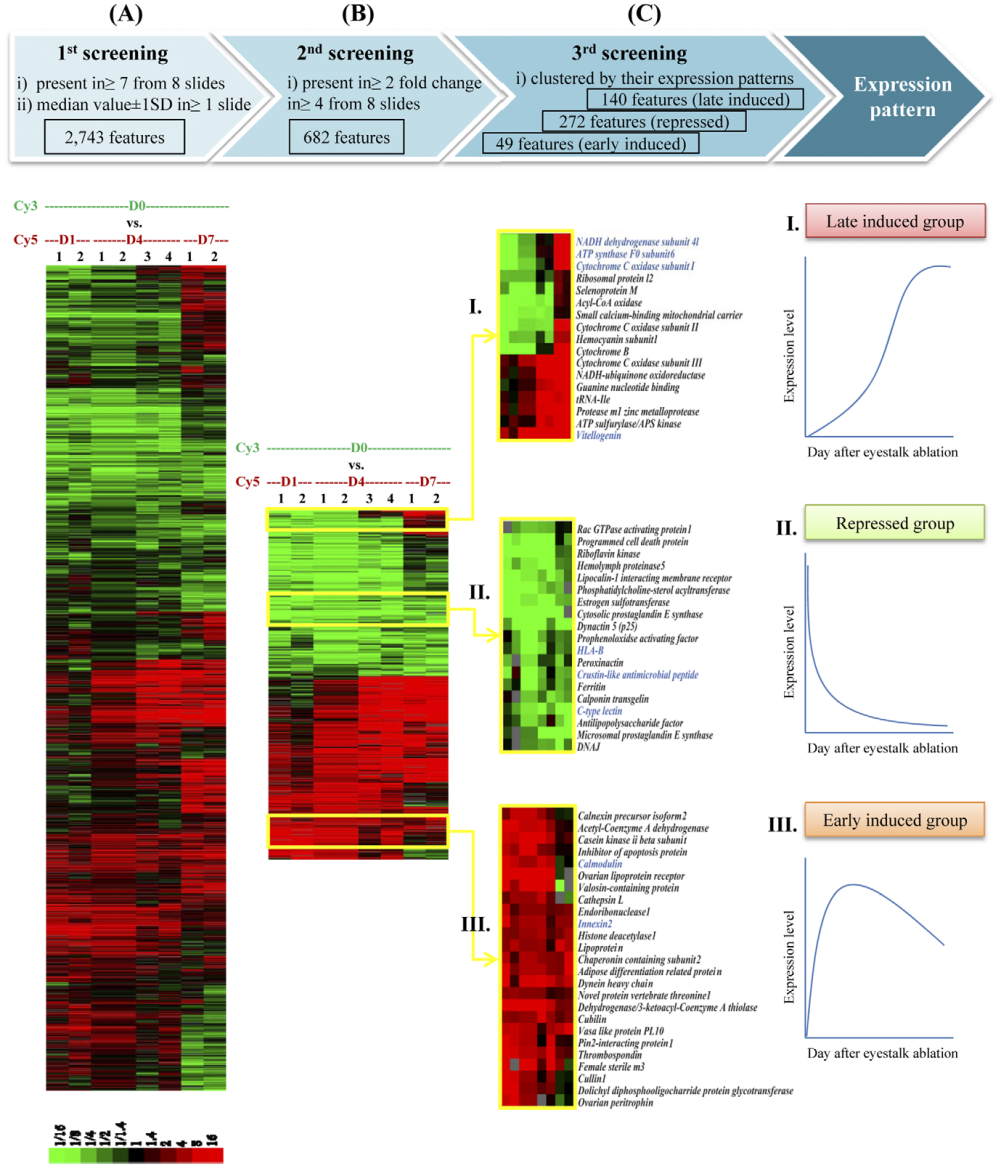
Gene expression analysis by cDNA microarray. Transcript levels in ovaries after eyestalk ablation at Days 1, 4 and 7 (D1, 4 and 7, respectively; Cy5 Red) were compared to those from non-ablated broodstock (D0, pooled from n = 7; Cy3 Green) using cDNA microarray. (A) A total of 2,743 transcripts present in at least 7 of the 8 microarrays with expression differences greater than median values ± SD (∼1.9-fold) in at least one arrays were selected. (B) Hierarchical clustering analysis of the 682 transcripts with expression difference greater than 2-fold change in at least 4 of the 8 microarrays. (C) Clusters I–III exhibit various differentially expressed patterns: Late induced (I), Repressed (II), and Early induced (III) groups. Transcripts in blue letters were further characterized by reverse- transcriptase quantitative PCR (RT-qPCR).

**Figure 3:**
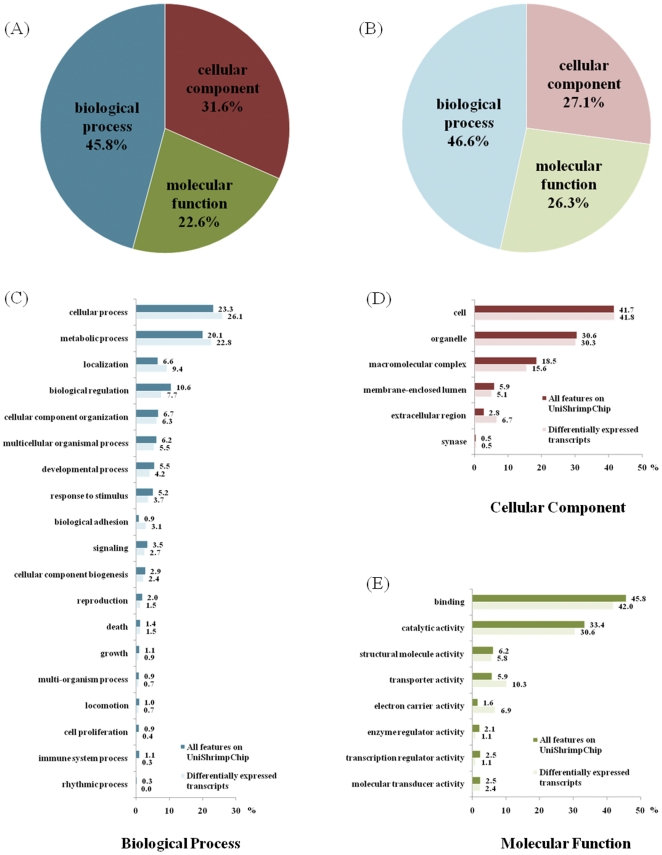
Gene Ontology (GO) annotation. Pie diagrams of the percentage distribution of sequences among the three principal GO categories were shown for (A) all 5,568 features on UniShrimpChip and (B) the 682 differentially expressed transcripts. Bar graph showed comparison between all features on UniShrimpChip (darker colored bar) and the differentially expressed transcripts (lighter colored bar) in each GO sub-category: (C) biological processes, (D) cellular components and (E) molecular functions.

To investigate transcriptomic changes as a result of the eyestalk ablation, a cDNA microarray, UniShrimpChip, was used to compare expression levels before and after the ablation in domesticated female black tiger shrimp broodstock (Fig. 2). From the microarray experiment, 2,743 transcripts present in at least 7 from 8 microarrays with expression levels ≥ median value±1SD (∼1.9-fold change) in at least 1 microarray were selected for further analysis (Fig. 2A). From these, 682 features with >2-fold change in at least 4 from 8 microarrays were considered differentially expressed and categorized based on Gene Ontology (GO; Fig. 3).

GO distribution of the 682 differentially expressed transcripts was compared to that of all 5,568 features on the UniShrimpChip (Fig. 3). According to the three GO categories, the highest numbers of the features belonged to the “biological process” (45.8% and 46.6% for the all array features and for the differentially expressed transcripts, respectively) followed by the “cellular component” (31.6% and 27.1%, respectively) and the “molecular function” (22.6% and 26.3%, respectively).

In the “biological process” category, various subgroups, such as cellular process, metabolic process, localization, biological regulation and biological adhesion, contained noticeably different distributions of the features between the array features and the differentially expressed genes (Fig. 3C).

Although the overall GO distribution of the differentially expressed transcripts (27.1%) was lower than of the array features (31.6%) for the “cellular components” category, the distributions among the subgroups in this category for the array features and the differentially expressed transcripts were mostly similar (Fig. 3D). In this category, transcripts involved in the cell subgroup were predominant, followed by those involved in organelle.

In the “molecular function” category, transcripts involved in binding activity were predominant, followed by those involved in catalytic activity (Fig. 3E). Among the subgroups in this category, distributions of the array features and the differentially expressed transcripts belonged to the transporter activity and electron carrier activity subgroups were the most distinct.

In addition to the GO analysis, the differentially expressed transcripts were grouped according to the similarity in gene expression profiles using Hierarchical cluster method (Fig. 2B). Three expression patterns of late induced (140 features), repressed (272 features) and early induced (49 features) transcripts were further examined as they include some transcripts with relevant functions (Fig. 2C). Cluster I contained genes whose expression levels were higher after the eyestalk ablation at the later days and was so called “Late induced group.” This transcript group includes a known marker for vitellogenesis such as *vitellogenin* and genes involved in energy metabolism mechanism such as *NADH dehydrogenase subunit4l*, *ATP synthase F0 subunit6*, *cytochrome C oxidase subunitI*, *II*, *III*, *cytochromeB*, and *acyl-CoA oxidase*. In contrast, genes in Cluster II were expressed lower after the ablation and categorized as “Repressed group.” This group includes genes involved in immune responses such as *HLA-B*, *crustin-like antimicrobial peptide*, *C-type lectin*, *antilipopolysaccharide factor*, and *hemolymph proteinase5*. Cluster III or “Early induced group” contained transcripts that were induced within a day after the eyestalk ablation. This group includes many known reproductive and hormonal genes (*ovarian lipoprotein receptor*, *thrombospondin*, *female steile m3* and *ovarian peritrophin*), genes involved in calcium signal transduction (*calmodulin* and *calnexin isoform2)* and genes involved in developmental processes (*innexin2* and *thyroid hormone receptor associated protein)*.

### Candidate pathways relevant to ovarian maturation

**Figure 4:**
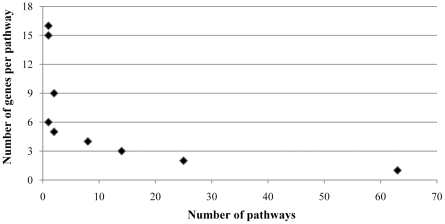
Distribution of number of genes assigned to predicted pathways from the KEGG database. A total of 117 pathways were retrieved from the mapping analysis of the 682 differentially expressed transcripts.

The differentially expressed transcripts were used to identify pathways in which the genes could be active after the eyestalk ablation. Of the 682 differentially expressed transcripts identified by the microarray analysis, 190 transcripts were automatically mapped on 117 different predicted pathways from the KEGG database, which features large numbers of biological pathways. The numbers of participated genes per pathway ranged from 1 to 16 (Fig. 4). A majority of the predicted pathways (63 pathways) were mapped with only a single participated gene, which cannot provide sufficient information in gene regulatory mechanism. Thus, the pathways with at least two participated genes were considered. According to the number of participated gene in each pathway, the pathways were ranked in the following order: ribosome, oxidative phosphorylation, RNA transport, protein processing in endoplasmic reticulum, glycolysis, fatty acid metabolism, ribosome biogenesis in eukaryotes, focal adhesion, insulin signaling pathway, tight junction pathway, GnRH signaling pathway, calcium signaling pathway, and progesterone-mediated oocyte maturation (Table 2).

**Table 2:** The pathways with the highest numbers of participated genes.

Pathways	Participated genes*	Day 1		Day 4		Day 7	
		Up	Down	Up	Down	Up	Down
Ribosome	16	3	13	4	12	5	11
Oxidative phosphorylation	15	6	9	8	7	10	5
RNA transport	9	5	4	5	4	4	5
Protein processing in endoplasmic reticulum	9	4	5	4	5	5	4
Glycolysis	6	2	4	2	4	3	3
Fatty acid metabolism	5	2	3	2	3	3	2
Ribosome biogenesis in eukaryotes	5	4	1	3	2	3	2
Focal adhesion	4	2	2	2	2	2	2
Insulin signaling pathway	4	2	2	2	2	1	3
Tight junction	4	2	2	1	3	1	3
GnRH signaling pathway	3	1	2	1	2	0	3
Calcium signaling pathway	2	1	1	1	1	0	2
Progesterone-mediated oocyte maturation	2	2	0	2	0	1	1

*only genes with differential expression level.

Considering the pathway with the highest number of participated genes, the ribosome pathway with 16 differentially expressed genes participated exhibited down-regulated expression patterns during the eyestalk ablation. The example genes in this pathway included *ribosomal proteins and elongation factor 1 alpha* (*EF-1α*). The oxidative phosphorylation pathway ranked second with 15 differentially expressed genes exhibiting later induction pattern at Days 4–7 after the ablation, and the participated genes included those encoding for protein subunits of the five enzyme complexes from electron transport chain such as *NADH dehydrogenase subunit4l*, *ATP synthase F0 subunit6*, *cytochrome C oxidase subunitI*, *II*, *III*, *VII*, and *cytochromeB* (Table 3).

**Table 3:** List of genes participated in the putative pathways analyzed by KASS.

Pathway	Genes participated in the putative pathways
**Ribosome**	*Ribosomal protein l23a*
	*Elongation factor 1-alpha, oocyte form*
	*Large subunit ribosomal protein l23e*
	*Ribosomal protein l26e*
	*Ribosomal protein l32*
	*Ribosomal protein s20*
	*Ribosomal protein l3*
	*Ribosomal protein s5e*
	*Ribosomal protein LP1*
	*Ribosomal protein s9*
	*Ribosomal protein l24e*
	*Ribosomal protein l31*
	*Ribosomal protein l44*
	*Ribosomal protein s19*
	*Ribosomal protein S30e fusion protein*
	*Ribosomal protein l27*
**Oxidative Phosphorylation**	*NADH dehydrogenase subunit1*
	*NADH dehydrogenase subunit4l*
	*NADH dehydrogenase subunit5*
	*NADH dehydrogenase (ubiquinone) 1 beta subcomplex3*
	*NADH-ubiquinone oxidoreductase sgdh subunit*
	*NADH dehydrogenase (ubiquinone) 1 beta subcomplex10*
	*CytochromeB*
	*ATP synthase F0 subunit6*
	*Cytochrome C-1*
	*Ubiquinol-cytochrome C reductase complex ubiquinone-binding protein qp-c*
	*Cytochrome C oxidase subunitI*
	*Cytochrome C oxidase subunitII*
	*Cytochrome C oxidase subunitIII*
	*Cytochrome c oxidase subunitVII*
***GnRH*** ** Signaling**	*Guanine nucleotide-binding protein G(q) subunit alpha*
	*Calmodulin*
	*Growth factor receptor-binding protein*
**Calcium Signaling**	*Guanine nucleotide-binding protein G(q) subunit alpha*
	*Calmodulin*
**Progesterone-mediated**	*Cell division cycle25*
**Oocyte Maturation**	*Calmodulin*

In addition to the top ranked pathways, three pathways (GnRH signaling pathway, calcium signaling pathway, and progesterone-mediated oocyte maturation pathway) were selected for mapping analysis using GenMAPP software because the early induced expression patterns of their participated genes after the ablation suggesting that these pathways might be potentially important as molecular responses to the eyestalk ablation. Moreover, these pathways have previously been known as a potential signaling pathway active during ovarian maturation in rats and crustaceans [16], [17].

**Figure 5:**
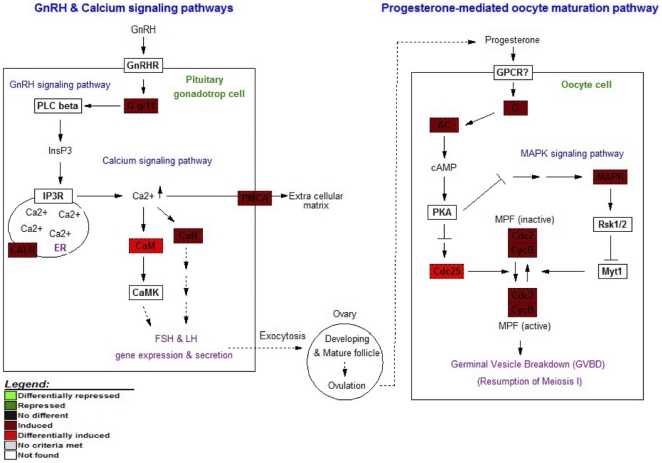
Putative pathways affected by the eyestalk ablation. The gonadotropin-releasing hormone (GnRH) signaling pathway, calcium signaling pathway, and progesterone-mediated oocyte maturation were identified as putative affected pathways containing early induced genes after the eyestalk ablation. The lighter red boxes represent the genes whose expression levels were induced more than 2 fold, whereas the dark red boxes represent the genes whose expression levels were induced less than 2 fold at Day 1 after the ablation. Abbreviation: GnRHR: gonadotropin-releasing hormone receptor; G q/11: guanine nucleotide-binding protein G(q) subunit alpha; PLC beta: phospholipase C beta; InsP3: inositol-1,4,5-triphosphate; IP3R: inositol-1,4,5-triphosphate receptor; CALR: calreticulin; ER: endoplasmic reticulum; Ca2+: Calcium ion; CaM: calmodulin; CaMK: calmodulin kinase; CaN: calcineurinB; PMCA: Ca2+ transporting ATPase; GPCR?: G protein-coupled receptor; G: guanine nucleotide-binding protein G(i) subunit alpha; AC: adenylate cyclase; PKA: cAMP-dependent protein kinase; Cdc25: cell division cycle25; Cdc2: cell division cycle2; CycB: cyclinB; MAPK: mitogen-activated protein kinase; Rsk1/2: p90 ribosomal S6 kinase; Myt1: membrane-associated tryrosine and threonine-specific cdc2 inhibitor kinase.

Therefore, the biological relationships among the three pathways were investigated together by visualizing the expression patterns of the participated gene expression (Fig. 5). A total of 11 genes involved in these signaling pathways and with expression data from microarray were *guanine nucleotide-binding protein G(q) subunit alpha (*G *q/11)*, *calreticulin (CALR)*, *calmodulin (CaM)*, *calcineurinB (CaN)*, *Ca^2+^ transporting ATPase (PMCA)*, *guanine nucleotide-binding protein G(i) subunit alpha (G)*, *adenylate cyclase (AC)*, *cell division cycle25 (Cdc25)*, *cell division cycle2 (Cdc2)*, *cyclinB (CycB)*, and *mitogen-activated protein kinase (MAPK)*. All of these transcripts in the putative pathways were up-regulated one day after the ablation.

**Figure 6:**
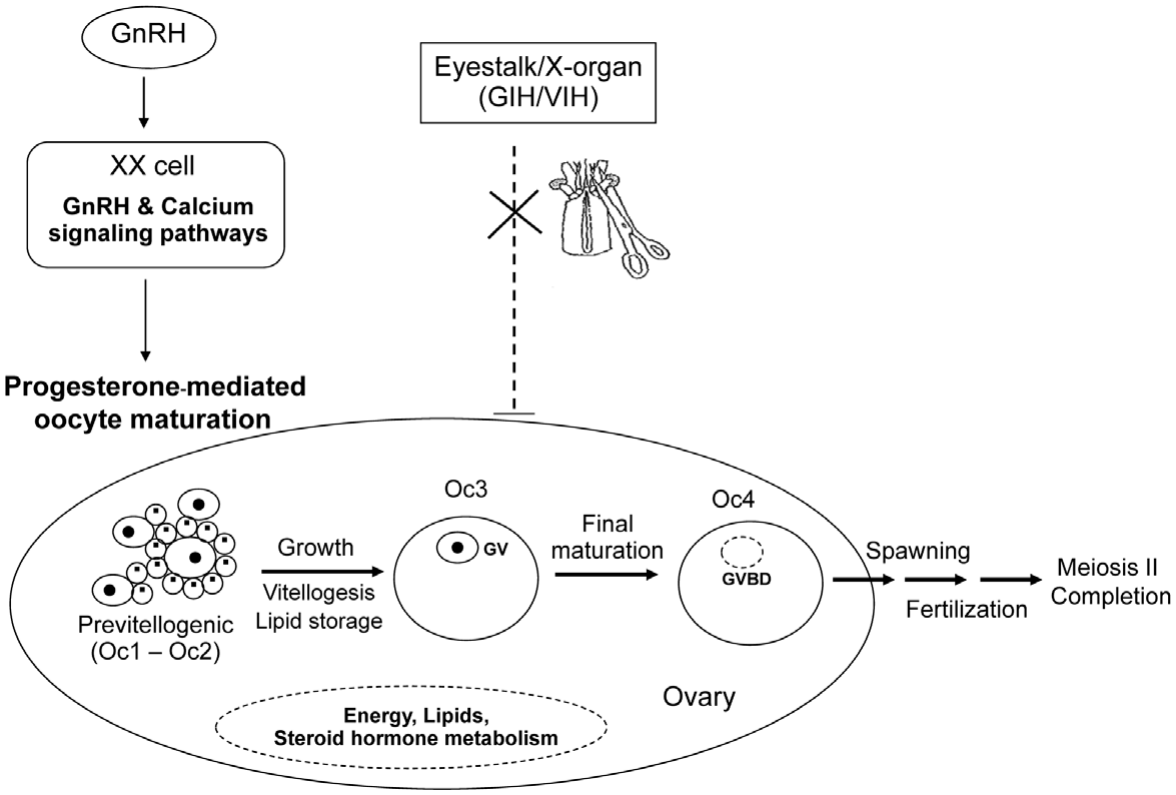
The proposed scheme to induce ovarian maturation by eyestalk ablation in *P. monodon*. Eyestalk ablation releases gonad- or vitellogenesis- inhibiting hormones (GIH or VIH) allowing a synthesis of yolk protein. GnRH activates the calcium signaling pathway resulting in increased levels of intracellular calcium ion through the GnRH signaling and calcium signaling pathways, which subsequently activates folliculogenesis, estradiol and progesterone production in ovary. Progesterone may then induce progesterone-mediated oocyte maturation process from oocyte stage 1 to 4; Oc1–4. “XX” indicates that this type of cell is not known in shrimp but it is pituitary gonadotrope cell in other organisms. Abbreviation: GnRH: gonadotropin-releasing hormone receptor; GIH: gonad inhibiting hormones; VIH: vitellogenesis inhibiting hormones; GV: germinal vesicle; GVBD: germinal vesicle breakdown.

By integrating gene expression data and retrieving biological pathway information affected by eyestalk ablation, major cellular pathway interactions in ovarian maturation of *P. monodon* were proposed (Fig. 6). The previtellogenic oocytes were induced early by activation of the gonadotropin-releasing hormone (GnRH) signaling pathway and the calcium signaling pathway, but inhibition of gonad- or vitellogenesis- inhibiting hormones (GIH or VIH) upon eyestalk ablation. Changes in signaling molecules of these biological pathways stimulated the release of progesterone leading oocyte maturation. The increased levels of transcripts including vitellogenin and energy-production genes seemed to drive the development of the previtellogenic to matured oocytes with germinal vesicle breakdown (GVBD) before spawning.

### Validation of gene expression by reverse-transcriptase quantitative PCR (RT-qPCR)

Based on the different gene expression patterns of microarray analysis, pathway mapping and gene functions potentially involved in reproductive maturation, gene expression patterns of 12 transcripts were analyzed using reverse-transcriptase quantitative PCR (RT-qPCR) (Table 4). Of these, nine genes were selected to represent the three gene expression patterns identified by microarray (Fig. 2C): Late induced (*NADH dehydrogenase*, *cytochrome C oxidase*, *ATP synthase F0*, and *vitellogenin)*, Repressed (*crustin-like antimicrobial peptide*, *C-type lectin*, and *HLA-B*), and Early induced (*innexin2*, and *calmodulin*). Since *calmodulin* was one of the differentially expressed transcripts with the early induced pattern and was mapped into calcium signaling pathway (Fig. 5), two additional genes (*calcineurinB* and *calmodulin kinase*) found in the same pathway were further examined to confirm the involvement of this pathway. Additionally, due to potentially relevant functions in reproductive maturation and the early induced expression pattern from the microarray, *thyroid hormone receptor associated protein* was also included in the RT-qPCR analysis.

**Table 4:** Primers used in Reverse-transcriptase Quantitative PCR (RT-qPCR) analysis.

Primer	Sequence	Size (bp)
***Late induced transcripts***		
*NADH dehydrogenase*	F: 5′ TAC TCT GTA GTT CGT GGA TGT GA 3′	191
	R: 5′ CGT GAA AAG AGC AAT GAA AGG TGG 3′	
*Cytochrome C oxidase*	F: 5′ CGA AAG AGG AGT GGG AAC TGG AT 3′	118
	R: 5′ TGA TGA GAC CCC TGC TAA ATG TAA 3′	
*ATP synthase F0*	F: 5′ GTC ATC ACA TAC GGG CTC AAC TC 3′	182
	R: 5′ GTC GTG TAT GAT TAC TCC AAC CAA 3′	
*Vitellogenin*	F: 5′ ATT CGG AAC GTG CAT TTG CTG CA 3′	188
	R: 5′ GTT CTC AAG CAT TGT GAC AGG ATT 3′	
***Repressed transcripts***		
*Crustin-like antimicrobial*	F: 5′ GAA TCG CTG CGG GCA TCC TCG GC 3′	244
*peptide*	R: 5′ AGA GAA AAT CAC TAC CCA AAA GA 3′	
*C-type lectin*	F: 5′ CAT CCC GAT TGT CCT CAC CTG TA 3′	217
	R: 5′ TTA CGG CTC CCC CAA CCC AGA AG 3′	
*HLA-B*	F: 5′ GCA GGA GTA GGA GAA ACG ACA GA 3′	150
	R: 5′ AGA AGG ATG CTC AAA GCC ACA GT 3′	
***Early induced transcripts***		
*Innexin2*	F: 5′ GGC ATC GCC AAC CCC AAC ATC CA 3′	164
	R: 5′ ACC AGC ATC TTT ACC TTA CCA CC 3′	
*Calmodulin*	F: 5′ GCA CCA TCA CCA CCA AGG AAC TG 3′	105
	R: 5′ TTA CCG TCA GCG TCC ACC TCG TT 3′	
***Reproduction-relevant transcripts***		
*Thyroid hormone receptor*	F: 5′ TAC AAC GAG TCC TGA GAA ACG GC 3′	105
*associated protein*	R: 5′ GGG AGT CAC CCA AGA GAG AGA AC 3′	
*Calcineurin B*	F: 5′ TTG ACA ACT CTG GCT CAC TAT CCG 3′	157
	R: 5′ ACA CTG AAC TGT GAC ATG CCT TGG 3′	
*Calmodulin kinase*	F: 5′ GAC CGA AGT GAT GGA AGC AAA AGT 3′	186
	R: 5′ ACA GAG AAG GAT GTA TGT GAT GAC 3′	
***Housekeeping gene***		
*Elongation factor-1α*	F: 5′ TTC CGA CTC CAA GAA CGA CC 3′	122
	R: 5′ GAG CAG TGT GGC AAT CAA GC 3′	

**Figure 7:**
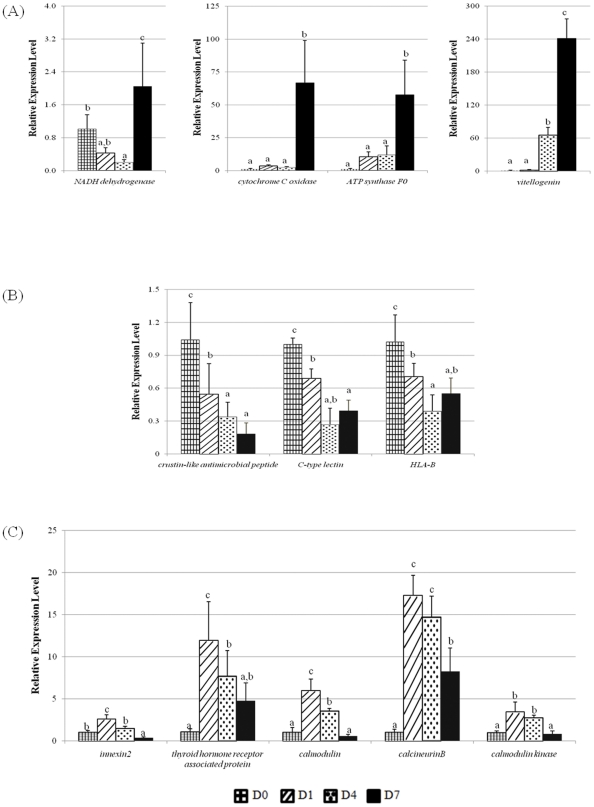
Reverse-transcriptase quantitative PCR (RT-qPCR) analysis. The relative expression levels of the selected transcripts in ovaries before (D0; *n* = 5) and after eyestalk ablation of *P. monodon* broodstock at Days 1, 4, and 7 (D1, D4, and D7, respectively; *n* = 5 each) were measured using RT-qPCR to confirm microarray results and identify other genes potentially involved in ovarian maturation via eyestalk ablation. A total of 12 transcripts were selected to represent three different expression patterns: (A) Late induced, (B) Repressed, and (C) Early induced groups. Different letters above each bar indicate significant difference (*P<0.05*; Tukey test).

For the late induced genes, the *NADH dehydogenase*, *cytochrome C oxidase*, *ATP synthase F0*, and *vitellogenin* transcripts exhibited significant increases in gene expression levels at Day 7 when compared to earlier time points and before eyestalk ablation (Fig. 7A). Among the four examined genes, at Day 7, the *vitellogenin* transcript level was dramatically increased by 240-fold from its initial level before the ablation (D0). Similarly, the *cytochrome C oxidase* and *ATP synthase F0* transcript levels were significantly expressed highest at Day 7 after the ablation with ∼58- and 67-fold increases, respectively. Although the induction of the *NADH dehydrogenase* transcript was ∼2 fold which was not as high as the other genes examined from this group, it was significantly induced at Day 7 (*P*<0.05).

For the repressed group, the *crustin-like antimicrobial peptide*, *C-type lectin*, and *HLA-B* transcripts were selected for RT-qPCR analysis. All three genes were significantly repressed at either Day 4 or Day 7 (*P*<0.05). The decreasing pattern of expression levels was in agreement with the microarray data. At Day 7, the expression levels of these transcripts were ∼2–5 folds lower than those before the eyestalk ablation (Fig. 7B).

The transcript levels of *innexin2*, *thyroid hormone receptor associated protein*, *calmodulin*, *calmodulin kinase*, and *calcineurinB* from the RT-qPCR analysis confirmed their early induction patterns determined by the microarray data as well (Fig. 7C). The expression levels were significantly induced to ∼3-, 8-, 4-, 17- and 12-fold, respectively, by one day after the eyestalk ablation (*P*<0.05). After the significant induction of these genes at Day 1, their expression levels were gradually reduced on the later days. At the end of the ablation experiment, some of the expression levels of these transcripts (*innexin2*, *calmodulin*, and *calmodulin kinase*) were even lower than their initial levels (D0).

## Discussion

Eyestalk ablation is commonly practiced to induce ovarian maturation in the black tiger shrimp farming [1]. This study aimed to understand effects of the eyestalk ablation in female domesticated broodstock at a molecular level by using cDNA microarray to rapidly screen for transcripts differentially expressed and using biological pathway information to reveal pathways potentially involved in the molecular response to the ablation.

In this study, based on the increased gonadosomatic index (GSI; ratio of gonad weight to body weight) and physiological changes from the bigger size and greener color of the ovaries, the unilateral eyestalk ablation evidently induced ovarian maturation within seven days. The GSI value is a good indicator of female reproductive maturation [18] in penaeid shrimp such as in *Penaeus setiferus* [19], *P. duorarum* [20], and *P. monodon* [5]. Based on the GSI values, physiological changes such as ovary color, and morphology of ovarian cells, ovarian maturation in penaeid shrimp can be categorized into four stages: pre-vitellogenic (undeveloped), vitellogenesis (developing), cortical rod (nearly ripe), and late cortical rod (ripe) [5]. From the result, by seven days after the eyestalk ablation, the ovaries were induced to mature to the late cortical rod stage.

From the microarray result, the eyestalk ablation in the female brooders altered expression levels of several important genes group. First and foremost, after the eyestalk ablation, the significant increase in the *vitellogenin* transcript level at the time when the color of the ovaries turned green and the GSI value reached the mature stage of ovary confirmed that the ablation led to vitellogenesis, which is the accumulation of a major yolk protein marking the start of ovarian development [21]. Vitellogenin has also been known as one of the most reliable markers correlated to ovarian maturation as its gene expression positively correlated to the ovarian maturation levels in *Marsupenaeus japonicus* [22] and in *P. monodon* [13].

To find potential biomarkers whose expression levels correlated to ovarian maturation levels, genes with similar expression patterns to that of the *vitellogenin* transcript were considered. It is interesting to find that genes whose functions relevant to energy metabolism, such as *NADH dehydrogenase*, *cytochrome C oxidase*, and *ATP synthase F0*, share similar gene expression patterns to that of the *vitellogenin* transcript. The induced expression of energy-related genes may imply that there is an increased flux of energy during the ablation. A previous study revealed that eyestalk ablation resulted in lower glucose levels in hemolymph of female *Litopenaeus vannamei* [23] and increasing energy requirement to maintain glucose level in hemolymph during gonadal maturity in *P. notialis* [24]. The increasing energy required after the ablation supports the up-regulation of genes important to the energy production process in our study. The elevated expression levels of these energy metabolism transcripts in this study may help improving farming practice by emphasizing the important of high energy source feed for shrimp broodstock during vitellogenesis. Vitellogenesis undoubtedly requires high energy to synthesize a female-specific lipoprotein called vitellogenin, which carries lipids and proteins for egg formation [25]. As most of lipid classes have a crucial role for energy metabolism [26], it is not surprising to see an increase in ovarian lipids level in maturing ovaries in *P. japonicus* [27]. Better ovarian development was also observed when feed was supplemented with lipid rich components [28], [29], such as polychaete [30], [31], [32], [33], [34]. Additionally, some previous reports showed that lipid composition of *P. monodon* is influenced by the lipid composition of its food [35], and phospholipid and PUFA are required for reproduction in *P. vannemei* [28], [36].

To see the immediate effect of eyestalk ablation, transcripts exhibiting early induction pattern after the ablation were examined through pathway mapping analysis. These transcripts participated in the GnRH signaling, calcium signaling, and progesterone-mediated oocyte maturation pathways. These activated pathways from our results agree with previous studies of induced oocyte maturation mechanism in penaeid shrimp [37], [38], [39], in human [40], and in African clawed frog [41]. For instance, it has been suggested that GnRH-like peptides were found in *P. monodon* [42] and other crustaceans such as *Marsupenaeus japonicus* [43] and *Macrobrachium rosenbergii* [44]. Moreover, eyestalk ablation has previously been found to induce ovarian maturation by inhibiting the GIH in *P. notialis* [24], and double-stranded RNA of GIH has been successfully used to reduce the *GIH* transcript level resulting in induction of ovarian maturation in *P. monodon* [11]. Therefore, the GnRH signaling pathway might exist and involve in the *P. monodon* ovarian maturation.

In other organisms and crustaceans, the GnRH pathway controlled through calcium levels via calmodulin and calnexin can regulate hormone release, and in turn, trigger lipoprotein transporters for vitellogenesis [16], [17]. Calnexin, similar to calrecticulin, is one of the calcium binding chaperones [45] involved in oogenesis in mammalian cells [46] by regulating GnRH receptor in human [16]. Calmodulin is a calcium binding protein involved in the regulation of steroidogenesis and oocyte maturation in rat [47], in amphibian [48], [49], in fish [50], and in crustacean [51] by activating calmodulin kinase [52] leading to the release of gonadotropins which in turn activate folliculogenesis, estradiol and progesterone production. Then, high level of progesterone synthesized and released by follicular cell is postulated to induce oocyte maturation through progesterone-mediated oocyte maturation pathway resulting in activation of maturation-promoting factor (MPF) consisting of cell division cycle2 and cyclinB. MPF has an important role in promoting oocyte maturation into previtellogenic stage. This molecular change presented in early induced transcripts could be supported by physiological growth of oocyte in *P. monodon* [53]. Although the actual molecular mechanism of the involvement of the GnRH signaling pathway in the ovarian maturation is not known, the results from this study may suggest that the eyestalk ablation perhaps induce ovarian maturation through the peptide hormone, GnRH, which activates various molecules such as G q/11, calmodulin, calmodulin kinase, and calcineurinB to increase intracellular calcium ion (Ca^2+^), and then progesterone is released to induce oocyte maturation in *P. monodon*. However, this hypothesis must be further examined to reveal the exact molecular mechanism to induce ovarian maturation.

Besides the genes participated in these pathways, *ovarian lipoprotein receptor*, *thrombospondin* (*TSP*), *ovarian peritrophin*, *innexin2* and *thyroid hormone receptor associated protein* (*TRAP*) were induced early after the ablation. Their highly induced expression levels and relevant function potentially serve as biomarkers for ovarian maturation. The highly induced *ovarian lipoprotein receptor* transcript level agrees with its high expression level during vitellogenesis in *P. monodon* [13], [54] and in *P. japonicus* [55]. In reproductive maturation, lipoproteins transport lipid source to gonad [25], [56]. *Thrombospondin* (*TSP*) and *ovarian peritrophin* involved in ovarian cell development as their expression levels were induced during oocyte maturation in *M. japonicus* [57], [58], in *P. semisulcatus* [59] and in *P. monodon* [13]. *Innexin2* encodes for a transmembrane protein found in epithelial cells [60] and functions as an intercellular communicator between soma cells and germ-line cells in oogenesis of *Drosophila malanogaster* [61]. *TRAP* encodes for a transcriptional co-activator binding to the nuclear receptor family that functionally involved in several mechanisms including androgen receptor signaling pathway, thyroid hormone receptor binding and vitamin D receptor binding [62], [63], which is essential for mammary gland development [64] and the progression and maintenance of embryonic development [65]. Therefore, it would not be surprising if TRAP would have a role in developmental processes during ovarian development in *P. monodon*.

Not only did the ablation significantly induced subsets of transcripts, it also repressed an important group of genes of immune-relevant genes. Indeed, eyestalk ablation has been previously reported to affect immune responses in *P. vannamei* when by altering the concentrations of several hemolymph metabolites important in modulating the immune response [23]. Similarly, in the Indian spiny lobster (*Panulirus homarus*), total hemocyte count (THC) and prophenoloxidase (PO) activity were increased within a short period of 2 hours and then decreased at 1 week after the ablation [66]. The increasing hormone gene expression levels in pre-mating processes resulted in the decreasing immune gene expression levels in *D. melanogaster* [67]. The changes of the immune gene expression levels in various organisms from the previous and our studies suggested a linkage between the immune response with the eyestalk ablation.

In summary, besides the well-known effects of the eyestalk ablation on physiological changes leading to ovarian maturation in the female black tiger shrimp, the ablation leads to several crucial transcriptomic changes. Several transcripts were identified to be potential biomarkers for ovarian maturation. With the power of high-throughput gene expression analysis using cDNA microarray and biological pathway information, several groups of transcripts and pathways were identified as putative mechanism resulted from the eyestalk ablation. The removal of eyestalk results in increasing of cell localization, transporter activity responded to hormonal control and calcium signaling pathways in an early stage. Induction processes stimulate vitellogenesis to occur using transporter and electron carrier molecules to increase energy production. These findings pinpoint several potentially important molecular mechanisms of the eyestalk ablation to be further examined. The results also help guiding farming practice to include high energy source in maturation feed formulation.

## Materials and Methods

### Animal collection and eyestalk ablation

Female domesticated *P. monodon* broodstock (14-month-old) were collected from the Shrimp Genetic Improvement Center (SGIC, Surat Thani, Thailand). Brooders were maintained at a biosecure station containing seawater pumped from the Gulf of Thailand at a salinity of 30 ppt with water temperature at around 27°C, and acclimated in tanks with 2 m in diameter for 3 weeks. Ovaries of the brooders were collected before (D0; *n = *7) and after unilateral eyestalk ablation for 1, 4, and 7 days (*n = *8 for each time point; Table 1 and Fig. 1A). All ovary samples were quickly frozen in liquid nitrogen and stored at −80°C until use.

### RNA extraction and cDNA labeling for hybridization

RNA samples were extracted from frozen ovaries using TRI-REAGENT according to manufacturer's instruction (Molecular Research Center, USA). Contaminating genomic DNA was removed by treatment with *DNase* I at 0.15 U/µg total RNA at 37°C for 30 min. The quality and quantity of the RNA were assessed on an agarose gel electrophoresis and NanoDrop (ND-8000) before subsequent experiments. DNase-treated RNA samples were converted to the first-strand cDNAs and labeled with aminoallyl-dUTP (aa-dUTP; Sigma) using a LabelStar Array kit (Qiagen). The aa-cDNA samples were cleaned up using a Microcon YM-30 filter (Millipore) and resuspended in 6 µl of 0.1 M sodium borate buffer (pH 8.7).

### cDNA microarray analysis

Construction of the *P. monodon* cDNA microarray (UniShrimpChip) used in these experiments was described previously [14], [15]. Briefly, the spotted cDNA microarray contains 5,568 cDNA features from 13 EST libraries of different *P. monodon* organs. UniShrimpChip was used to investigate effects of eyestalk ablation by comparing expression levels of ovaries before and after the ablation (Table 5). A reference sample (Cy3) was from ovary RNA samples pooled from eyestalk-intact domesticated broodstock (pooled from 7 individuals, D0, GSI = 1.1±0.2%), whereas Cy5 sample was from ovary RNA of individual broodstock after eyestalk ablation at Day 1 (*n* = 2, GSI = 1.2, 1.2%), Day 4 (*n* = 4, GSI = 1.5, 1.7, 3.8, 4.2%), and Day 7 (*n* = 2, GSI = 6.8, 7.4%).

**Table 5:** Summary of microarray experiment.

Green (Cy3)	Red (Cy5)
Before eyestalk ablation	After eyestalk ablation
D0 (pooled, *n* = 7; GSI* = 1.1±0.2%)	Day 1: D1 (individual, *n* = 2; GSI* = 1.2, 1.2%)
	Day 4: D4 (individual, *n* = 4; GSI* = 1.5, 1.7, 3.8, 4.2%)
	Day 7: D7 (individual, *n* = 2; GSI* = 6.8, 7.4%)

*Gonadosomatic Index (GSI) = % gonad weight/body weight.

The slides were post-processed to block unused surfaced using a BSA-based method and microarray procedures were performed as previously described [13]. Briefly, the aa-cDNA was fluorescently labeled with Cy3- or Cy5- dyes (GE Healthcare) at room temperature for 1 h. The unincorporated dye was removed using a Microcon YM-30 filter and the purified probe was resuspended in 6 µl of TE. The Cy3- and Cy5- samples were mixed together in a hybridization buffer (50% formamide, 0.1% SDS, 5× SSC, 0.2 µg/µl of yeast tRNA and 0.2 µg/µl of salmon sperm DNA) in 30 µL of total reaction volume and denatured at 95°C in the dark for 3 min before applying onto the post-processed slide with a glass cover slip (22×32 mm, Deckglasser) for each experiment. Hybridization was performed overnight (∼16 hr) in a sealed hybridization chamber (CORNING) at 42°C. The hybridized arrays were immersed into a pre-warmed washing buffer I (2× SSC and 0.1% SDS) to remove a cover slip before transferred to wash in the same buffer for 5 min with gently shaking. The arrays were washed in a washing buffer II (0.2× SSC) and a washing buffer III (0.05× SSC) for 5 min each at a room temperature with gently shaking. The arrays were then dried by centrifugation at 1,000 rpm for 5 min at room temperature (Allegra X-22 R, Beckman coulter) and scanned on an AXON GenePix 4000B microarray scanner (Axon Instruments, Molecular Devices, Sunnyvale, CA, USA).

### Analysis of microarray data

Microarray data were extracted using GenePix Pro version 6.1 software. Relative gene expression levels were obtained by comparing the amounts of transcript present in the experimental samples (Cy5-labeled sample) to a reference sample (Cy3-labeled sample). Only spots with intensities greater than one standard deviation (SD) above the background intensity were further analyzed.

The processed data were normalized within each array by global normalization method, and across arrays, using the Aroma package [68] (available from http://www.maths.lth.se/help/R/aroma/), run in R project environment (http://cran.r-project.org). The microarray data is MIAME compliant and the raw data has been deposited in a MIAME compliant database (NCBIs Gene Expression Omnibus, http://www.ncbi.nlm.nih.gov/geo/, with GEO accession number GSE 29025 at [69]). From all features on the microarray, only transcripts present in 7 from 8 microarrays and with expression changes ≥ the median expression values±1SD in one of the arrays were considered further. The differentially expressed transcripts were selected from those with ≥2-fold changes than the median expression±1SD in 4 of 8 microarrays. The differential expressed transcripts were grouped together according to their expression patterns by a Hierarchical clustering analysis using the Cluster 3.0 software and visualizing gene expression profile using the TreeView 1.6 software [70].

### Mapping of candidate genes to pathways

The gene expression data from the microarray analysis were used to examine potential roles of these transcripts in physiological and biochemical processes by linking them with the Kyoto Encyclopaedia of Genes and Genomes (KEGG) database using two software programs called KEGG Automatic Annotation Server (KAAS) (http://www.genome.jp/kegg/kaas/) [71] and Gene Map Annotator and Pathway Profiler (GenMAPP) [72]. Briefly, the KASS was used to automatically link the microarray data to existing pathways from the KEGG database. The parameter on KASS was set for ESTs annotation with the bi-directional best hit (BBH) method. Later, the expression values of the differentially expressed genes and the retrieved information of pathways were integrated and visualized by using GenMAPP software comparing expression levels across the eyestalk ablation time course from Days 1–7.

### Reverse-transcriptase quantitative PCR (RT-qPCR)

RT-qPCR experiment was performed for 12 transcripts (*NADH dehydrogenase*, *cytochrome C oxidase*, *ATP synthase F0*, *vitellogenin*, *crustin-like antimicrobial peptide*, *C-type lectin*, *HLA-B*, *innexin2*, *calmodulin*, *thyroid hormone receptor-associated protein*, *calcineurinB*, and *calmodulin kinase*) (Table 4). *Elongation factor 1 alpha* (*EF-1α*), a housekeeping gene, was used as an internal control. The expression study was performed using templates from individual eyestalk-intact broodstock (D0, *n* = 5), and eyestalk-ablated broodstock at Days 1, 4 and 7 (*n* = 5 for each group). Each reaction was performed in a 20-µl total volume containing 2× iQ™ SYBR® Green Supermix (Bio-Rad), 200 ng of first strand cDNA template, and 0.2 µM of primer pair. Cycling parameters were 95°C for 2.5 min; followed by 40 cycles of 95°C for 30 sec, 58°C for 20 sec, and 72°C for 30 sec. The specificity of PCR products was confirmed by melting curve analysis performed from 55°C-95°C with a continuous fluorescent reading with every 0.5°C increment. The relative expression levels of copy number of the target gene to that of a housekeeping gene *EF-1α* in different sample groups were statistically tested by ANOVA followed by Duncan's new multiple range test or Tukey test (*P*<0.05) [15].
